# Complete Genome Sequence of Halomonas meridiana Strain Eplume2, Isolated from a Hydrothermal Plume in the Northeast Pacific Ocean

**DOI:** 10.1128/MRA.00330-20

**Published:** 2020-05-14

**Authors:** Yu Kurihara, Shingo Kawai, Ayano Sakai, Josephine Galipon, Kazuharu Arakawa

**Affiliations:** a Institute for Advanced Biosciences, Keio University, Tsuruoka, Japan; b Faculty of Policy Management, Keio University, Fujisawa, Japan; c Faculty of Environment and Information Studies, Keio University, Fujisawa, Japan; d Systems Biology Program, Graduate School of Media and Governance, Keio University, Fujisawa, Japan; e Exploratory Research Center on Life and Living Systems, National Institutes of Natural Sciences, Okazaki, Japan; University of Southern California

## Abstract

Halomonas meridiana strain Eplume2 (ATCC BAA-804) is a Gram-negative bacterium isolated from hydrothermal plume seawater in the Northeast Pacific Ocean at a depth of 2,000 m. Here, we report the complete genome sequence of this strain, which has a total size of 4.12 Mbp and a 56.6% G+C content.

## ANNOUNCEMENT

*Halomonas* is a genus of Gram-negative haloalkaliphilic proteobacteria. Some of its members are isolated from hypersaline environments, and most require high salinity for growth. This group of bacteria is resilient to various environmental stresses and is expected to become a reliable model organism for bioindustrial applications (1), including for the production of useful secondary metabolites (2, 3). Halomonas meridiana was first isolated from the hypersaline lakes of Vestfold Hills, Antarctica, in 1990 (4). Here, we report the complete genome of *H. meridiana* strain Eplume2 (ATCC BAA-804), isolated from hydrothermal plume seawater in the Northeast Pacific Ocean.

*Halomonus meridiana* strain Eplume2 (ATCC BAA-804) was isolated by Kaye et al. from hydrothermal plume water of the Juan de Fuca Ridge in the Northeast Pacific Ocean at a depth of 2,000 m (5). The strain was obtained from the original discoverer, J. Z. Kaye, in lyophilized form and is identical to the strain available at the ATCC (BAA-804; https://www.atcc.org/products/all/BAA-804.aspx). The dried sample was rehydrated in SW10 culture solution and spread onto an SW10 agar plate. A single colony of this strain was cultured overnight using SW10 medium at 37°C, and the total genomic DNA was extracted and purified using a Genomic-tip 20/G kit following the manufacturer’s protocol (Qiagen). The DNA library for long-read sequencing was prepared using a rapid barcoding kit (SQK-RBK004; Oxford Nanopore Technologies), and sequencing was performed on the GridION device with a FLO-MIN106 flow cell (Oxford Nanopore Technologies). Illumina sequencing was performed for error correction using a KAPA HyperPlus kit for library preparation, and the library was sequenced as 75-bp single-end reads with a NextSeq 500 sequencer with high-output mode and 75 cycles (Illumina). After Nanopore sequencing, a total of 108,276 reads (*N*_50_, 15.8 kbp) were obtained, and reads longer than 10,000 bp, corresponding to around 60× coverage, were used for assembly using Canu v.1.8 (6). The assembled sequence was manually circularized by deleting the overlapping end. Error correction was performed by mapping all 52 million raw Illumina reads with the Burrows-Wheeler Aligner (BWA) v.0.7.11 (7), and then polishing was performed for four rounds using Pilon v.1.23 (8). Assembly completeness was checked with Benchmarking Universal Single-Copy Orthologs (BUSCO) v.1 (9) on the gVolante server (10) and resulted in 100% completeness of all 40 core genes. The obtained complete genome sequence was functionally annotated using the DDBJ Fast Annotation and Submission Tool (DFAST) pipeline (11). The annotated genome has a total length of 4,118,995 bp with a 56.6% G+C content, harboring 3,941 coding sequences (CDSs), 60 tRNAs, and 18 rRNAs. All software was run using default parameters.

According to the annotation results, *H. meridiana* strain Eplume2 carries numerous genes involved in sulfur metabolism, including a homolog of the sulfite reductase *cysI* (HMEPL2_05320), a well-known enzyme responsible for the conversion of SO_3_^2−^ (sulfite) to H_2_S (hydrogen sulfide) (12). This is in agreement with the initial report describing this strain as positive for H_2_S production (5). The search for analogs of H_2_S-responsive genes in halophilic species could lead to the development of biosensors for H_2_S in hypersaline environments.

### Data availability.

The complete genome sequence of *H. meridiana* strain Eplume2 has been deposited in DDBJ under the accession number AP022869 and in the Sequence Read Archive (SRA) under the BioProject number PRJNA613015.
